# Supplementary material for: Involvement of prelimbic cortex neurons and related circuits in the acquisition of a cooperative learning by pairs of rats
Source: Cogn Neurodyn. 2024 May 3;18(5):2637–58. doi: 10.1007/s11571-024-10107-y (PMC11564578; doi:10.1007/s11571-024-10107-y)
Supplement: Supplementary file 1 — Supplementary file1 (PDF 832 KB) [file 11571_2024_10107_MOESM1_ESM.pdf]

Cognitive Neurodynamics
Research Article

Involvement of prelimbic cortex neurons and related circuits in the acquisition of a cooperative learning by pairs of rats

A.R. Conde-Moro[1], F. Rocha-Almeida[1], E. Gebara[2], J.M. Delgado-García[1], C. Sandi [2], A. Gruart[1]

1. Division of Neurosciences, Pablo de Olavide University, Seville-41013, Spain
2. Laboratory of Behavioral Genetics, Brain Mind Institute, School of Life Sciences, École Polytechnique Fédérale de Lausanne, Lausanne, Switzerland

Supplementary material

5 figures and their figure legends

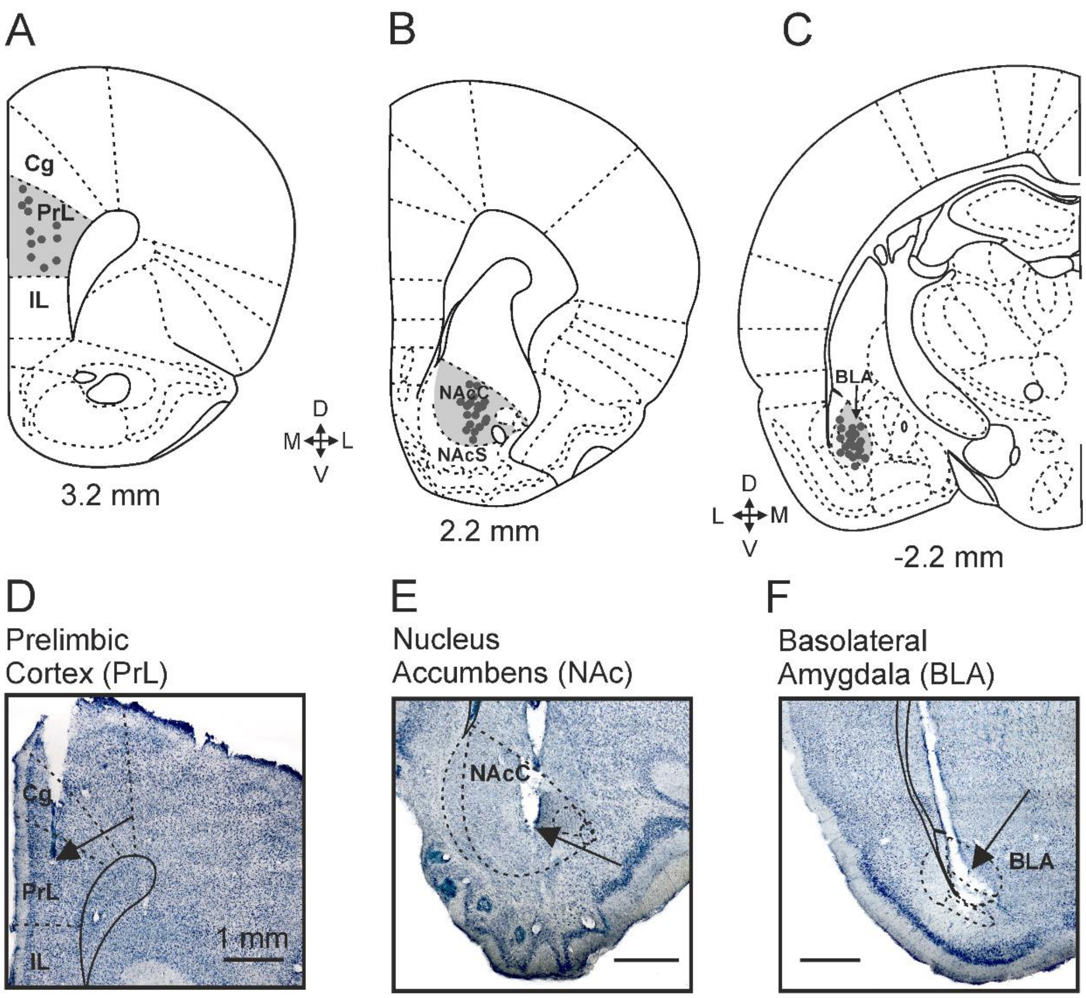

Supplementary figure 1. A-C. Diagrams representing the final locations of each electrode in the three areas recorded. **A**, each rat was implanted with two sets of recording electrodes aimed at the right PrL cortex (3.24 anterior and 0.5 mm lateral to bregma, and 2.5 mm from the surface). **B**, each rat was implanted with two sets of recording electrodes aimed at the right NAc (2.2 mm anterior and 1.5 mm lateral to bregma, and 6.5 mm from the surface). **C**, a third set of recording electrodes was aimed at the left BLA (2.28 posterior and 5 mm lateral to bregma, and 7.5 mm from the surface). **D-I**, photomicrographs of the brain regions of interest showing the final location of the recording electrodes. The tissue was dyed following the Nissl technique. The arrows point to the scar left by the electrode in the tissue.

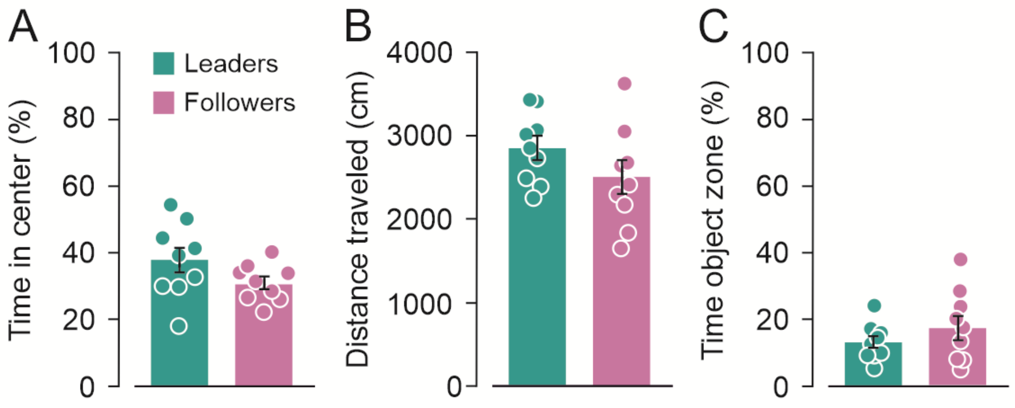

Supplementary figure 2. Open field test (groups 2 and 3). **A**, percentage of time spent in the center and border areas of the OFT. Although *leader* rats spent more time in the open arms —more anxiogenic— than *followers*, there was high variability among subjects, and the difference between groups was not significant (One-way ANOVA, F = 2.58, p = 0.12). **B**, distance traveled. Again, *leader* rats, in general, traveled more distance than *followers*, but the differences were not significant (One-way ANOVA, F = 2.58, p = 0.12). **C**, time spent in the novel object zone. Non-significant differences were found between the percentage of time that *leader* and *follower* rats spent in the object zone (One-way ANOVA, F = 1.10, p = 0.30).

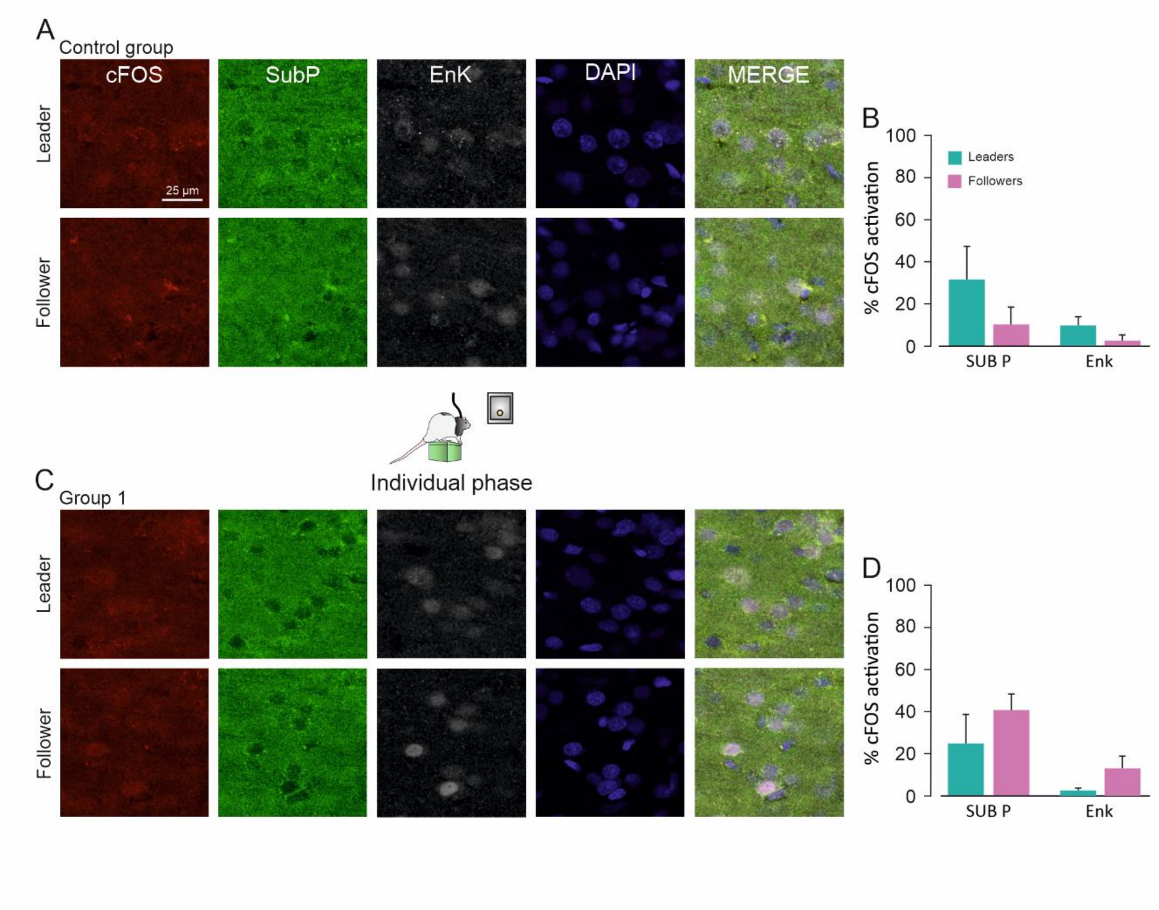

Supplementary figure 3. c-FOS expression in D1- and D2-containing cells in the PrL cortex of the individual (group 1) and control group. **A**, **C,** Same configuration as in Fig. 4A-C. The photomicrographs corresponding to the control group, which did not participate in the experiments, are shown in A. The photomicrographs in **C** correspond to Group 1, in which rats were trained for the individual phase until reaching the criterion. **B**, **D**, The percentage of c-FOS activation in each group of cells was quantified. *Predicted follower* rats presented higher percentages of c-FOS expression than *predicted leaders*, but non-significant differences were found both in the level of activation of D2 cells during the individual phase (**D**, One-way ANOVA, F = 0.68, p = 0.42) and in the control group (**B,** One-way ANOVA on ranks, H = 0.02, p = 0.93). Non-significant differences were found both in the level of activation of D2 cells for *predicted leaders* and *predicted followers* during the individual phase (**D**, One-way ANOVA on ranks, H = 2.41, p = 0.11) and in the control group (**B**, One-way ANOVA, F = 1.85, p = 0.20).

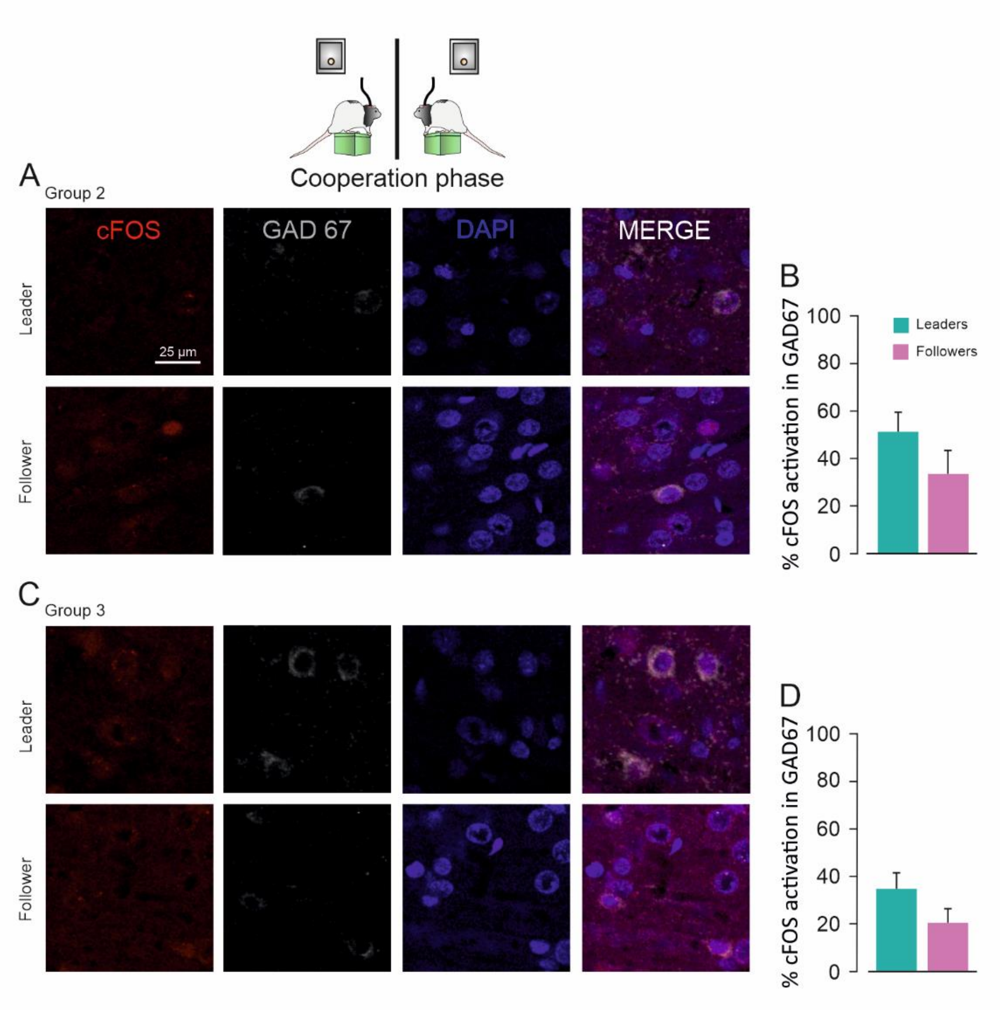

Supplementary figure 4. c-FOS expression in PrL GABAergic cells of cooperation groups (groups 2 and 3). Coronal sections from the four groups of animals were labeled for c-FOS (red channel), DAPI (blue channel), and mouse anti-GAD67 (gray channel) antibody for staining GABAergic cells of the PrL cortex. The photomicrographs corresponding to group 2, in which rats were trained to cooperate until reaching the criterion, are shown in **A**. The photomicrographs in **C** correspond to group 3, which completed the 10 days of the task. The percentage of c-FOS activation in GABAergic cells was quantified (**B-D**). Although *follower* rats in groups 2 and 3 showed a tendency for activation of GABAergic cells greater than that of *leaders*, no significant differences were found (**B**, One-way ANOVA, $F = 2.50$, $p = 0.12$) and 3 (**D**, One-way ANOVA, $F = 1.62$, $p = 0.22$).

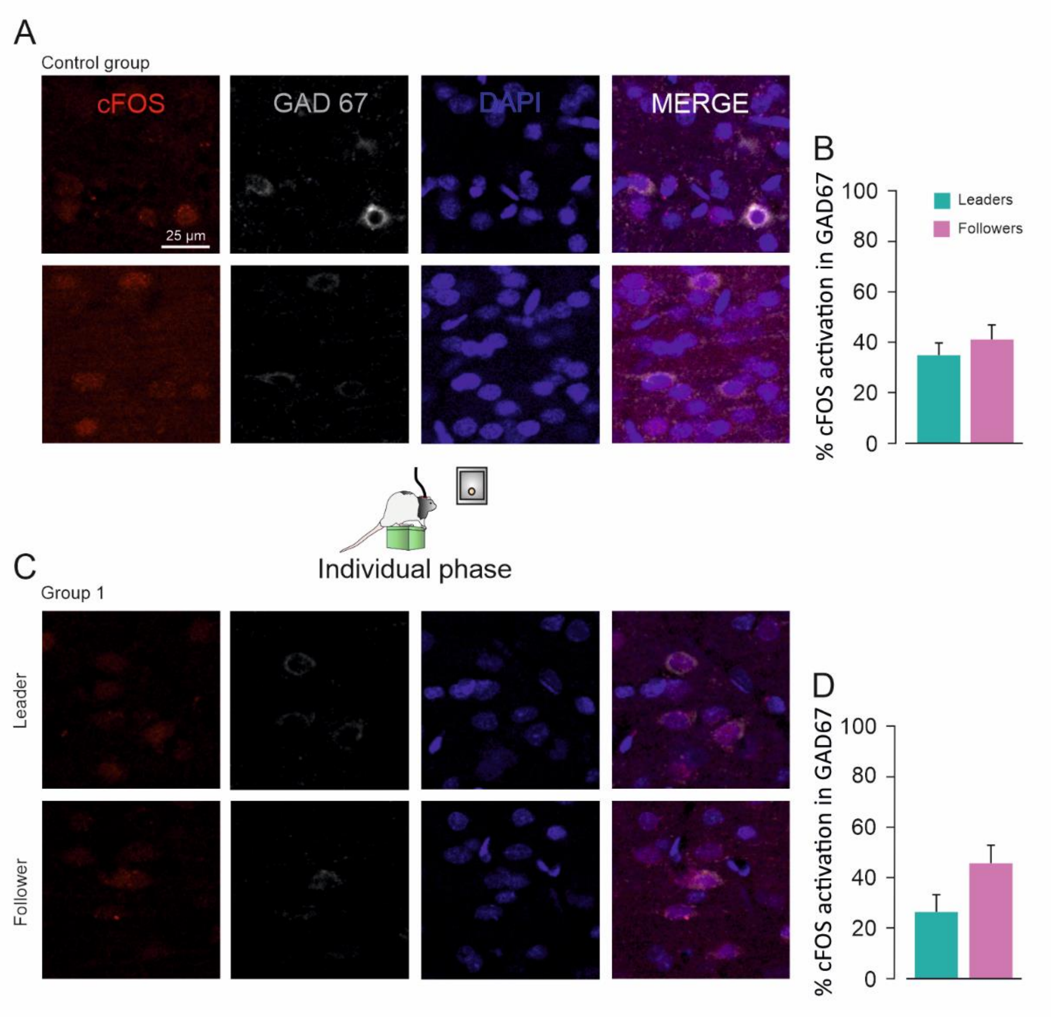

Supplementary figure 5. c-FOS expression in PrL GABAergic cells of control group and group 1. Coronal brain sections were labeled for c-FOS (red channel), DAPI (blue channel), and mouse anti-GAD67 (gray channel) antibody for staining GABAergic cells of the PrL cortex. The photomicrographs corresponding to the control group, which did not participate in the experiments, are shown in **A**. The photomicrographs in **B** correspond to group 1, which performed the individual task until reaching criterion. The percentage of c-FOS activation in GABAergic cells was quantified (**B-D**). Although *predicted follower* rats in group 1 showed a tendency for higher activation of GABAergic cells, non-significant differences were found between *predicted leaders* and *predicted followers* in both of these groups (**B**, One-way ANOVA, $F = 2.15$, $p = 0.16$; **D**, One-way ANOVA, $F = 0.49$, $p = 0 .49$).
